# Influence of Hydroxyapatite Nanospheres in Dentin Adhesive on the Dentin Bond Integrity and Degree of Conversion: A Scanning Electron Microscopy (SEM), Raman, Fourier Transform-Infrared (FTIR), and Microtensile Study

**DOI:** 10.3390/polym12122948

**Published:** 2020-12-10

**Authors:** Rana S Al-Hamdan, Basil Almutairi, Hiba F Kattan, Noura A. Alsuwailem, Imran Farooq, Fahim Vohra, Tariq Abduljabbar

**Affiliations:** 1 Department of Restorative Dental Sciences, College of Dentistry, King Saud University, 60169, Riyadh 11545, Saudi Arabia; ralhamdan@ksu.edu.sa (R.SA.-H.); balmutairi@ksu.edu.sa (B.A.); 2 Preventive Dental Science Department, Princess Nourah bint Abdulrahman University, Riyadh 11545, Saudi Arabia; Hfkattan@pnu.edu.sa; 3 School of Dentistry, Riyadh Elm University, Riyadh 12395, Saudi Arabia; Nouralsuwailem@gmail.com; 4 Faculty of Dentistry, University of Toronto, Toronto, ON M5S 1A1, Canada; imran.farooq@mail.utoronto.ca; 5 Department of Prosthetic Dental Science, College of Dentistry, King Saud University, Riyadh 11545, Saudi Arabia; tajabbar@ksu.edu.sa; 6 Research Chair for Biological Research in Dental Health, College of Dentistry, Riyadh 11545, Saudi Arabia

**Keywords:** nano-hydroxyapatite, dentin adhesive, microtensile bond strength, Scanning Electron Microscopy (SEM), Fourier Transform-Infrared (FTIR), degree of conversion, Micro-Raman spectroscopy

## Abstract

An experimental adhesive incorporated with different nano-hydroxyapatite (n-HA) particle concentrations was synthesized and analyzed for dentin interaction, micro-tensile bond strength (μTBS), and degree of conversion (DC). n-HA powder (5 wt % and 10 wt %) were added in adhesive to yield three groups; gp-1: control experimental adhesive (CEA, 0 wt % HA), gp-2: 5 wt % n-HA (HAA-5%), and gp-3: 10 wt % n-HA (HAA-10%). The morphology of n-HA spheres was evaluated using Scanning Electron Microscopy (SEM). Their interaction in the adhesives was identified with SEM, Energy-Dispersive X-ray (EDX), and Micro-Raman spectroscopy. Teeth were sectioned, divided in study groups, and assessed for μTBS and failure mode. Employing Fourier Transform-Infrared (FTIR) spectroscopy, the DC of the adhesives was assessed. EDX mapping revealed the occurrence of oxygen, calcium, and phosphorus in the HAA-5% and HAA-10% groups. HAA-5% had the greatest μTBS values followed by HAA-10%. The presence of apatite was shown by FTIR spectra and Micro-Raman demonstrated phosphate and carbonate groups for n-HA spheres. The highest DC was observed for the CEA group followed by HAA-5%. n-HA spheres exhibited dentin interaction and formed a hybrid layer with resin tags. HAA-5% demonstrated superior μTBS compared with HAA-10% and control adhesive. The DC for HAA-5% was comparable to control adhesive.

## 1. Introduction

Dentin is a calcified tissue that forms the majority of the tooth structure; it is comprised of microscopic channels called dentinal tubules and an intra-tubular matrix [1,2]. Dentin hypersensitivity (DH) results from the exposure of these tubules due to erosion, abrasion, attrition, abfraction, and dental caries [3,4,5]. To restore dental caries, multiple restorative materials are available; however, dental composites are among the most popular and widely indicated [6]. These dental composites are polymers combined with inorganic fillers that mimic natural teeth aesthetically, although their long-term mechanical and chemical stability is debatable [7]. The failure rates of composite ranges between 15% and 50%, which commonly occurs due to secondary caries, surface wear, marginal discrepancies, and fractures [8]. Moreover, it is suggested that while bonding to enamel is easily achieved due to mechanical interlocking, the bonding of adhesive with dentin is more puzzling (due to the lower inorganic content) [9]. Furthermore, the depth of dentin tissue has a direct impact on the bond strength, and decreased bond strength could be observed in deeper dentin compared with superficial dentin [10].

Biomaterials have the ability to control stem cell behavior and their development for regenerative purposes [11]. In an earlier study, it was reported that hydroxyapatite (HA) nanoparticles of various sizes could promote stem cell differentiation with smaller sized HA particles being more effective [12]. The local microbiome under certain conditions can cause local inflammation [13], and here, the role of biomaterials again becomes important. Biomaterials that are tested for various parameters such as cell adhesion, cell support, and cell differentiation do better under in vivo conditions in terms of preventing diseases and promoting regeneration [14]. In order to improve the properties of dental resins, the incorporation of nanoparticles including metal oxides, fluorides, and antimicrobial agents in the polymer matrix was introduced [15,16,17]. A similar nanoparticle in the form of a non-toxic, chemically stable, and bioactive material is HA [18]. HA constitutes the majority of the inorganic part of the tooth tissue, and owing to HA’s admirable remineralization properties, its use for different dental applications has increased recently [19]. In the presence of the partial demineralization of the collagen matrix, the presence of HA particles could ensure remineralization by the precipitation of calcium and phosphate ions (provided by HA crystals) [20].

Adhesion is the property of a material that is directly dependent on the quality of the polymer formed, and dentin adhesives stimulate bonding between the treated dentin and the resin composites by linking the hydrophilic primer and hydrophobic composite resin [21,22]. Since dental adhesive comes in direct contact with the tooth, the incorporation of HA could stimulate effective and durable remineralization in teeth with demineralized dentin [23]. In addition, the inclusion of nano-HA (n-HA) particles in the adhesive resin applied to dentin could positively enhance the strength and structure of the tooth, along with improving the mechanical properties of the resin adhesive [24]. Adhesives with remineralization potential can trap matrix metalloproteinases (MMPs) in crystals, fossilizing MMPs and cathepsins in the dentin tissues, thus protecting collagen from degradation [20]. It is narrated that mechanical properties of dental composites are critical for its longevity, and the inclusion of HA particles in the adhesive has shown to bring enhancement in the adhesive’s bulk mechanical properties [21]. However, n-HA addition to the dentin adhesive may compromise its dentin penetration, dentin bond integrity, and degree of conversion (DC) [25].

In a study by Sadat-Shojai et al., it was suggested that HA nanorods potentially biomineralize the tooth by bonding between COOH, OH, and NH_2_ of the collagen and OH of HA particles for remineralization at the dentin–resin interface [26]. In another study, the addition of 7 wt % of HA nanorods improved the bond strength of adhesive to tooth dentin [27]. However, in a study by Leitune et al., the addition of higher content of n-HA particles showed a significant loss of shear bond strength of resin adhesive to dentin [21]. Therefore, the specific amount of HA added in the adhesive that could result in optimum bond integrity, strength, and DC is still debatable. It is hypothesized that the incorporation of an increasing content of n-HA will compromise the bond strength and degree of conversion of experimental dentin adhesive. Therefore, the purpose of the study was to synthesize an experimental adhesive incorporated with different n-HA particles concentrations and analyze its dentin interaction, micro-tensile bond strength (μTBS), and DC.

## 2. Materials and Methods

### 2.1. Synthesis of Experimental and HA Resin Adhesives

Employing the methods from the study by Ye et al., the experimental adhesive was synthesized [28]. A combination of monomers including bisphenol A glycol dimethacrylate (BisGMA), triethylene glycol dimethacrylate (TEGDMA), 2-hydroxyethyl methacrylate (HEMA), and ethyl 4- dimethylamino benzoate and camphorquinone (Esstech Inc., Essington, PA, USA) were used. The formulation included a mixture of 50% Bis-GMA, 25% TEGDMA, and 25% HEMA (60%) by weight with ethanol as a solvent (30%—m/m). First, 0.5% (n/n) ethyl 4-dimethylamino benzoate and 0.5% camphorquinone photo-initiators were incorporated according to the monomer moles. In addition, as an electron initiator, 1.0% (n/n) diphenyliodonium hexafluorophosphate (DPIHP) was also added to the adhesive mixture. The mixture was prepared in a three-necked flask with a magnetic stirrer and condenser (SA300; Sansyo, Tokyo, Japan). To prevent photo-polymerization, the prepared adhesive was kept in an isolated dark chamber covered with foil. This adhesive was regarded as control experimental adhesive (CEA).

Nano-spheres of HA (hydroxyapatitie, Sigma Aldrich, St. Louis, MO, USA) were obtained and silanized. In order to enhance the adhesion of n-HA to the adhesive, 5% silane was added to the solvent of 95% acetone for silanization. Silanized n-HA spheres were added in 10% and 20% concentrations (m/m) to the control adhesive to produce HA adhesive 5 wt % (HAA-5%) and HAA 10 wt % (HAA-10%). To achieve the homogenization of n-HA spheres in the adhesive components, the dispersion of HA using sonication in a centrifuge was performed. The formulated adhesives were stored at 37 °C for 24 h for allowing solvent evaporation. The adhesives were stored at 4°C and were to be used in 20 days due to having a limited shelf life.

### 2.2. Characterization of HA and Synthesized Adhesive

The morphology of n-HA spheres was evaluated using Scanning Electron Microscopy (SEM). The presence of n-HA spheres within the experimental adhesives was identified with SEM, Energy-Dispersive X-ray spectroscopy (EDX), and Micro-Raman spectroscopy. EDX identified the elements and chemical groups within the adhesives. Prior to assessment, the HA adhesive was placed on a glass slide and photo-polymerized using a light-curing unit (Eliphar S10; 600–800 mW·cm^−2^ output; 3M ESPE, St. Paul, MN, USA) for 20 s. Polymerized specimens were mounted on aluminum stubs and sputter-coated with gold for 100 s (Baltec sputter, Scotia, NY, USA). SEM evaluations were performed at different magnifications (FEI Quanta 250, Scanning Electron Microscope, Hillsboro, OR, USA), at 10 kV accelerating voltage.

### 2.3. Tooth Preparation and Bonding Procedure

Ninety extracted teeth were cleansed using an ultrasonic scaler (Superior Instruments Co, New York, NY, USA) and were treated with chloramine trihydrate solution (Merck, Darmstadt, Germany) for 48 h. Teeth were mounted in orthodontic resin (Opti-Cryl, South Carolina, Columbia) at the cement–enamel junction (CEJ) level within 15 mm (height) sections of polyvinyl pipes (4 mm) and stored in distilled water. Tooth dentin was exposed by removing occlusal enamel with a slow speed diamond saw (Buehler Isomet 2000 Precision saw, Lake Bluff, IL, USA). Dentin was conditioned with 35% phosphoric acid (Ultra etch Econo Kit- Optident- Yorkshire, UK) for 10 s followed by washing and drying with cotton pellets. Thirty teeth each were treated with CEA (Gp:1, control), HAA-5% (Gp:2), and HAA-10% (Gp:3) adhesives. Adhesives were dispensed on a paper pad and using a micro-brush smeared on a dentin surface with agitation for 10 s followed by air thinning for 3 s. Following another similar adhesive application, polymerization was done with a light-curing device (Eliphar S10; 600 mW·cm^−2^ output; 3M ESPE, St. Paul, MN, USA) for 20 sec at 10 mm distance. For each specimen, a resin composite build-up was performed on the bonded adhesive using a resin mold and metal condenser in 2 mm increments (Filtek Universal; 3M ESPE, St. Paul, MN, USA). The composite build-up was light cured for 20 s each side after the removal of excess. All bonded specimens in each adhesive group were stored in distilled water for 24 h at 37 °C. Twenty specimens in each adhesive group (CEA, HAA-5%, and HAA-10%) were used for the μTBS testing. Five (out of ten remaining) specimens in each group were assessed for bond integrity of the resin–dentin interface using SEM, and the last five bonded specimens in each adhesive group were utilized for Micro-Raman spectroscopy analysis.

### 2.4. Micro-Tensile Bond Strength (μTBS)

Prior to specimen sectioning, 10 bonded specimens in each adhesive group (CEA, HAA-5%, and HAA-10%) were thermo cycled (TC) in water baths at 5 °C and 55 °C for 30 s each and 5 s dwelling time (THE-1100, SD Mechatronik GmbH, Germany). However, the other 10 specimens remained non-thermocycled (NTC) in each adhesive group and were stored in distilled water for 1 week. The sectioning of all bonded specimens in the study was performed with a slow speed diamond saw (Buehler Isomet 2000 Precision saw, Lake Bluff, IL, USA) to form 1 mm × 1 mm, composite–dentin bonded beams. Six beams from each tooth were used for μTBS assessment (sixty) in each adhesive subgroup (CEA-TC, CEA-NTC, HAA-5%-TC; HAA-5%-NTC, HAA-10%-TC, HAA-10%-NTC). Bonded beams were secured to the micro-tensile tester (Bisco Inc., Richmond, VA, USA) jaws with cyanoacrylate (Superglue, Minneapolis, MN, USA) and were assessed under tension at a crosshead speed of 0.5 mm/minute until failure. Failure modes were evaluated among the groups, classified as adhesive, cohesive, and mixed types using a digital microscope (Hirox KH 7700, Tokyo, Japan). Means and standard deviations of μTBS among the groups were analyzed using ANOVA and a post hoc multiple comparisons test.

### 2.5. Micro-Raman Spectroscopy of Dentin–Adhesive Bonded Specimen

Five bonded specimens in each adhesive group were assessed using Micro-Raman. Using a Raman spectrometer (ProRaman-L Analyzer; TSI, Shoreview, MN, USA) and Raman microscopic software (Ramanreader), Raman spectra were identified. The specimen positioning was adjusted to target dentin, n-HA spheres, and the interface, and correction for dark counts was done to obtain adequate signals. By using a laser beam with 0.9 objective lens and 600 mW power, the interface was observed. Specimens were divided into zones of interest (1 mm sections), and 3 scans of 60 s were performed for each zone. The details of the spectra were obtained between the range of 800 cm^−1^ and 2000 cm^−1^ with noise filtration.

### 2.6. SEM Observation of Bonded Adhesive/Dentin Interface

Bonded beam specimens of CEA, HAA-5%, and HAA-10% (*n* = 5) were polished (Beuhler Polisher, Lake Bluff, IL, USA) and cleaned in an ultrasonic bath (Bandelin Digital- Sigma-Aldrich Darmstadt, Berlin, Germany) for 4 min. Bonded beams were further conditioned at the interface with 35% phosphoric acid gel (Ultra etch Econo Kit- Optident- Yorkshire, UK) for 10 s and washed with distilled water (15 s). Specimens were immersed in 5.25% of sodium hypochlorite (NaOCl) solution (5 min) and washed. An ethanol treatment at 80%, 90%, and 100% concentration was performed to desiccate the specimens. The specimens were mounted on aluminum stubs and sputter-coated with gold under vacuum (Baltec SCD sputter, Scotia, NY, USA) and visualized at multiple magnifications under SEM (FEI Quanta 250, Scanning Electron Microscope, Hillsboro, OR, USA) at 10 kV voltage.

### 2.7. Degree of Conversion (DC)

Employing transmission Fourier Transform-Infrared Spectroscopy (FTIR) the degree of conversion (DC) of the adhesives was assessed. Adhesives including, CEA, HAA-5%, and HAA-10% were evaluated before and after polymerization. Homogenized adhesive was smeared on the potassium bromide disc of the spectroscope (Shimadzu, Kyoto, Japan). The absorbance peaks for the C–C double bonds were recorded for the uncured resin, as the adhesives remained in contact with the FTIR sensors (Thermo Scientific Nicolet iS20 FTIR spectrometer, Waltham, MA, USA). The adhesive resins were polymerized using a dental light curing unit for 40 s, and the FTIR peaks were recorded again. Using a standardized baseline method [29], the C–C (aromatic) reference peaks (1607 cm^−1^) and C=C (aliphatic) absorbance peak (1638 cm^−1^) were obtained. The FTIR spectra were observed between 400 and 4000 cm^−1^ for determining the DC. The conversion rate for adhesives was identified by calculating the ratios of (C=C and C–C) absorbance intensities (percentage of unreacted double bonds) prior to and post photo-polymerization using the below equation [30].

DC = [1 − (C_aliphatic_/C_aromatic_)/(U_aliphatic_/U_aromatic_)] × 100%

where

C_aliphatic_ is 1638 cm^−1^ absorption peak of polymerized resin,
C_aromatic_ is 1607 cm^−1^ absorption peak of polymerized resin,
U_aliphatic_ is 1638 cm^−1^ absorption peak of unpolymerized resin and
U_aromatic_ is 1607 cm^−1^ absorption peak of unpolymerized resin.

## 3. Results

### 3.1. SEM/EDX Outcomes

**Figure 1:**
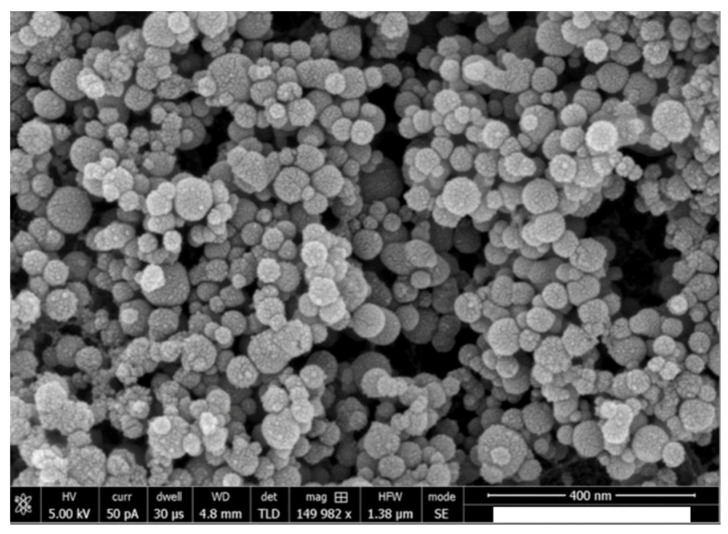
The morphological shape and size of the nanoparticles is depicted. It is noted that the size of the nano-hydroxyapatite (n-HA) particles are in nanoscale with a mean average size of <100 nm in diameter.

**Figure 2:**
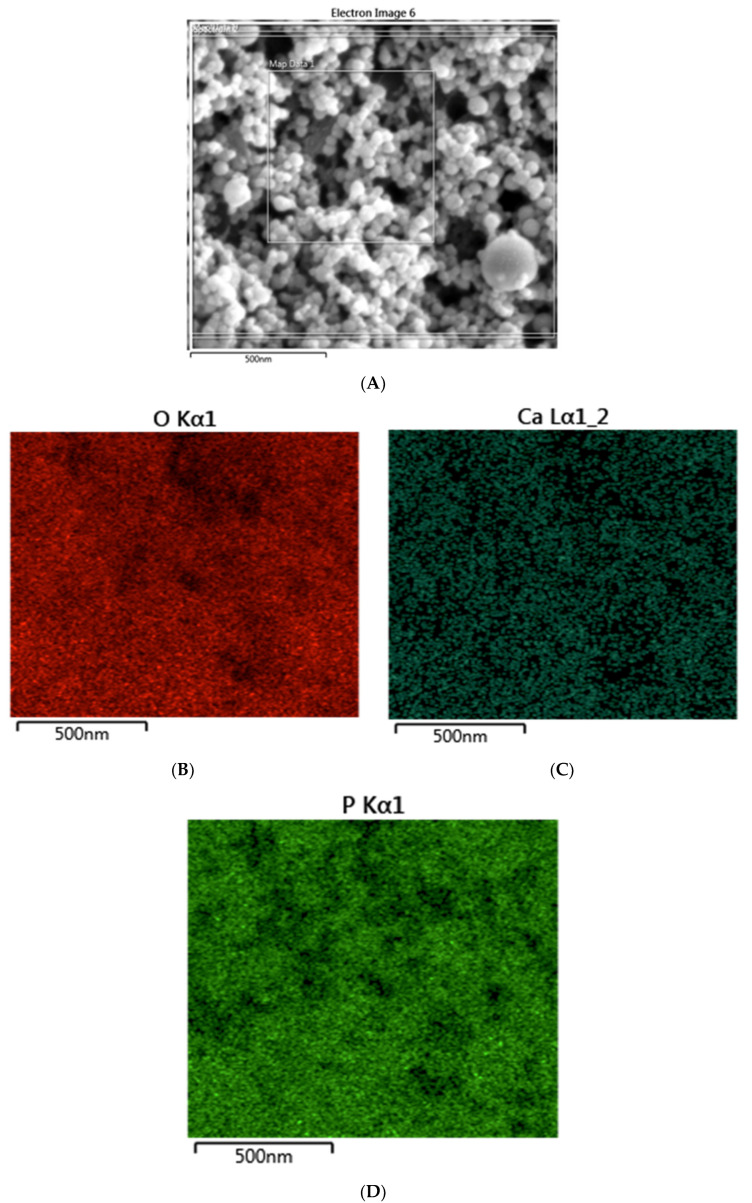
Energy-Dispersive X-ray (EDX) elemental mapping of the n-HA nanoparticles (**A**) showing evidence of oxygen (**B**), calcium (**C**), and phosphorus (**D**) in the hydroxyapatite samples.

**Figure 3:**
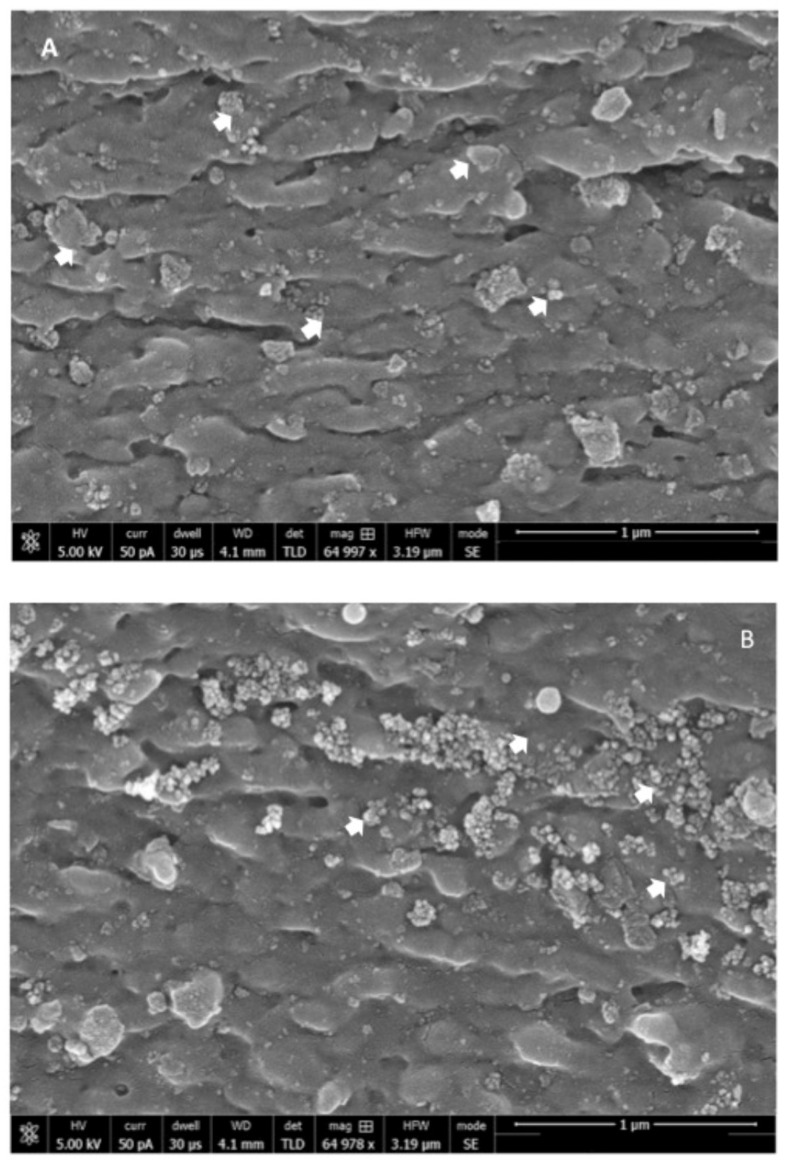
(**A**) Distribution of n-HA (white arrows) along the resin matrix showing homogenous dispersion of the low percentage weight (5 wt %) hydroxyapatite nanoparticles. (**B**) Distribution of n-HA (white arrows) in the resin matrix, showing dispersion of high percentage (10 wt %) with agglomerations.

**Figure 4:**
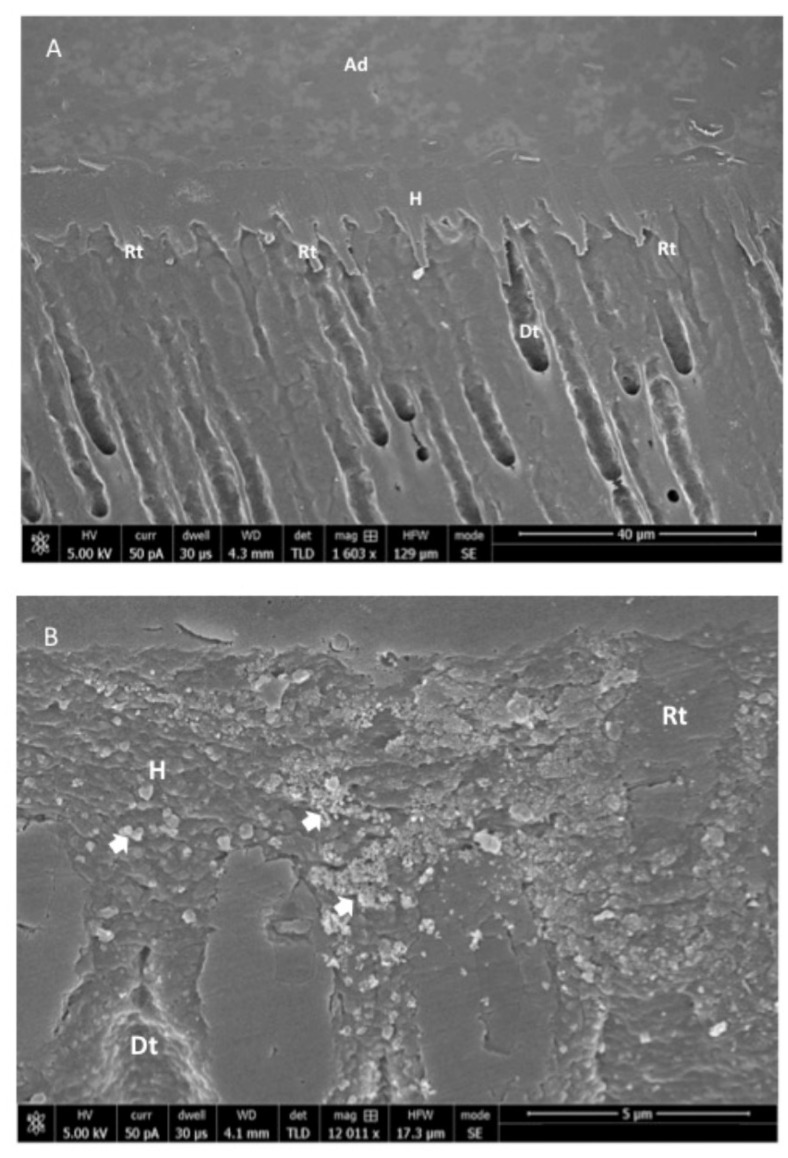
(**A**) Field emission scanning electron microscopic (FE-SEM) micrograph of resin-bonded sample showing the formation of a hybrid layer and integration between dentin and modified experimental gp-2: 5 wt % n-HA (HAA-5%) adhesive. Ad: adhesive, H: hybrid layer, Rt: resin tags; Dt: Dentin tubule. (**B**) Magnified image of the hybrid layer showing widely distributed n-HA particles within the hybrid layer (HAA-5%). Ad: adhesive, H: hybrid layer, Rt: resin tags; Dt: Dentin tubule.

On SEM analysis, the n-HA powder demonstrated an average powder size of <100 nm (Figure 1). With regard to the morphology, the n-HA powder particles were spherical in shape without any coarse edges (Figure 1). The EDX mapping analysis revealed the presence of oxygen, calcium, and phosphorus in the 5 wt % (Gp: 2) and 10 wt % (Gp: 3) HA samples (Figure 2). It was also observed that upon the mixing of n-HA powder in the experimental adhesive, a uniform distribution showing a homogenous dispersion of HA particles in the adhesive for HAA-5% (Figure 3A) and HAA-10% with agglomerates (Figure 3B) was observed. The Field Emission (FE)-SEM micrograph demonstrated the formation of a hybrid layer, and an admirable integration was observed between the dentin and HAA-5% (Figure 4A). Upon further magnification, widely distributed n-HA particles were seen in the newly formed hybrid layer as well (Figure 4B).

### 3.2. Microtensile Bond Strength (μTBS)

The μTBS MPa values (Mean ± SD) observed for the samples (both TC and NTC) of three groups in this study along with their respective failure modes are shown in Table 1. It can be observed that for the NTC samples, the HAA-5% group had the greatest mean μTBS values (32.50 ± 3.47) followed by CEA (27.36 ± 2.80) and HAA-10% (24.41 ± 3.72) groups. For the TC samples, the same pattern of μTBS values as observed with NTC samples was detected, and HAA-5% again had the greatest values (25.31 ± 3.84). On an intergroup comparison for NTC samples, the differences observed for CEA (27.36 ± 2.80) compared with HAA-5% (32.50 ± 3.47) and HAA-5% (32.50 ± 3.47) compared with HAA-10% (24.41 ± 3.72) were statistically significant (*p* < 0.01). For the intergroup comparison of TC samples, only the differences observed between CEA (21.71 ± 3.85) and HAA-10% (17.60 ± 4.36), and HAA-5% (25.31 ± 3.84) matched with HAA-10% (17.60 ± 4.36) were statistically significant (*p* < 0.01). On intragroup comparison for TC and NTC samples, differences between all groups were statistically significant (*p* < 0.01). Overall, if the μTBS values of TC and NTC specimens are compared, the NTC samples revealed higher mean values than the TC samples. There was no clear distribution pattern observed for the failure modes observed for the three groups; however, the majority of the failures observed were of adhesive type. Adhesive failure depicts a failure in adhesion with fractures, which are not observed in dentin or the resin [31]. In our study, although adhesive type failures were the most common, among them, the smallest percentage of adhesive-type failures was observed in NTC-HAA-5% followed by NTC-HAA-10%.

### 3.3. FTIR Analysis

**Figure 5:**
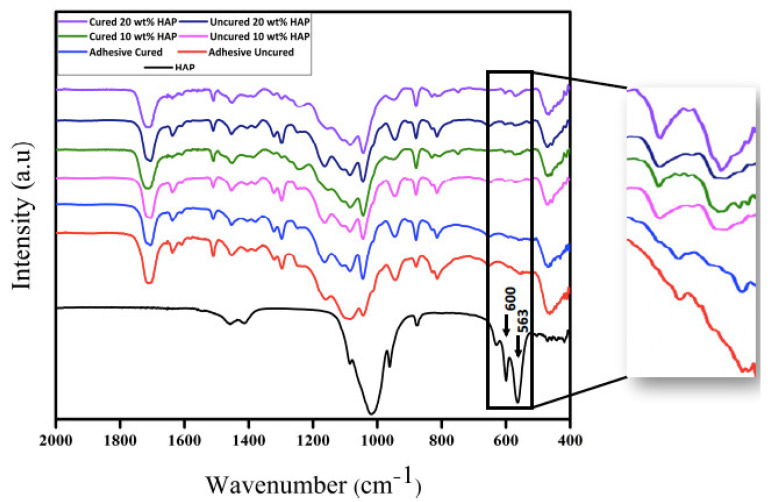
Fourier Transform-Infrared (FTIR) spectra of the n-HA particles, unmodified and modified experimental adhesives having low (5 wt %) and high (10 wt %) percentage of n-HA. The characteristic peaks at 563 and 600 cm^−1^ showing evidence of the presence of hydroxyapatite in the uncured and cured dentin adhesive.

The representative FTIR spectra of n-HA particles, CEA, HAA-5% (cured and uncured), and HAA-10% (cured and uncured) are shown in Figure 5. The characteristic peaks at 563 and 600 cm^−1^ show the evidence of the presence of apatite in the HAA. The apatitic phosphate groups usually demonstrate a characteristic split band at 560 and 600 cm^−1^, as observed for HAA in our study [32]. In addition, the presence of apatite could also be confirmed by the presence of a sharp single phosphate band [33], observed at 1040 cm^−1^ for HAA in our study.

### 3.4. Micro-Raman Spectroscopy Outcomes

**Figure 6:**
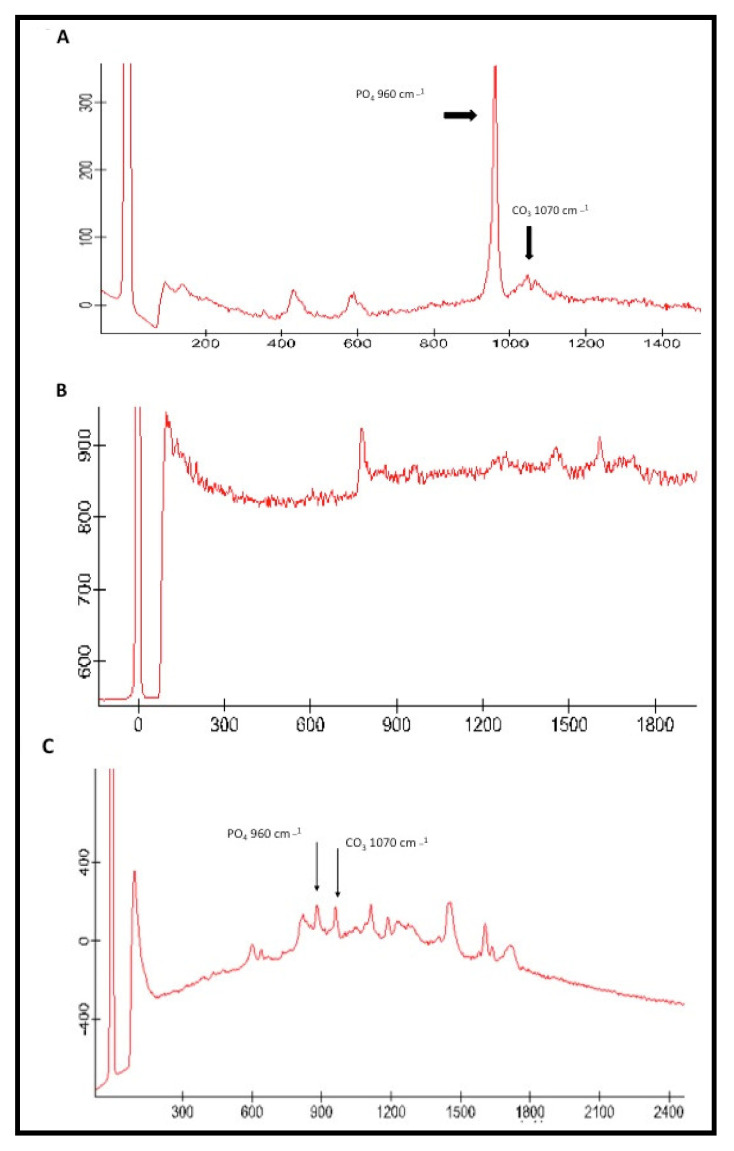
(**A**) Typical Raman spectra of the fabricated hydroxyapatite showing chemical groups identified by micro Raman analysis that could be identified as phosphate (υ1 PO_4_ ≈ 960 cm^−1^) and carbonate (CO_3_ ≈ 1070 cm^−1^). (**B**) Raman spectra of control adhesive and (**C**) HA-modified adhesive. The inclusion of HA nanoparticles in the adhesive is evidenced by the spectrum at 960 cm^−1^ for phosphate and 1070 cm^−1^ for carbonate.

The representative Micro-Raman spectra of n-HA particles, CEA, and HAA-5% is shown in Figure 6A–C, respectively. The phosphate and carbonate groups could be identified in Figure 6A at 960 cm^−1^ and 1070 cm^−1^ correspondingly. For CEA, the presence of phosphate and carbonate groups is not clear (Figure 6B). The Micro-Raman spectra of HAA-5% group again demonstrates the presence of the phosphate group at 960 cm^−1^ and carbonate group at 1070 cm^−1^ (Figure 6C).

### 3.5. Degree of Conversion (DC)

The highest DC was observed for the group CEA (62.3 ± 5.2) followed by HAA-5% (58.1 ± 4.8) and then HAA-10% (51.8 ± 5.6). The intergroup DC result comparisons were significantly different (*p* < 0.01), for groups CEA and HAA-10%; and HAA-5% equated with HAA-10% respectively (Table 2). No statistically significant (*p* > 0.01) results were observed when the DC results for CEA were compared with HAA-5% groups.

## 4. Discussion

The present study investigated the effect of inclusion of n-HA spheres and its different content levels (5 wt % and 10 wt %) in an experimental resin adhesive on its bond strength and DC. Based on the outcomes, 5 wt % n-HA showed higher μTBS and comparable DC to controls. However, μTBS and DC of 10 wt % n-HA adhesive (HAA-10%) was significantly lower than control and 5 wt % n-HA (HAA-5%) adhesive. Therefore, the hypothesis was partly rejected. A multitude of explanations including HA particle size, opacity, chemical composition, and adhesive viscosity can be attributed to the current observations.

Dental adhesives are critical in the clinical success of restorations, as a compromised res-adhesive bond is associated with microleakage, DH, and secondary caries development [9]. The use of nanoparticles as reinforcing filler improves the mechanical, physical, and working properties of dental composites, as opposed to macro or micro-sized fillers [34]. The researchers have incorporated various therapeutic ions including silver nanoparticles (AgNPs) [35], zirconium oxide–titanium dioxide (ZrO_2_-TiO_2_) nanoparticles [36], copper-doped bioactive glass nanoparticles (CuBGn) [37], and n-HA to improve the properties of adhesives [38]. The n-HA offers superior biocompatibility to act as a source of calcium and phosphate, in addition to possessing a strong ability to bond with tooth proteins [38,39]. It is also evidenced that n-HA particles are a better source of free calcium ions, which are required for remineralization [39]. These benefits of n-HA encouraged its incorporation to the experimental dental adhesive and its further influence on resin–dentin bond integrity, DC, and μTBS.

The μTBS test was employed in the present study, as it shows better discriminative competence [40], precise estimation of bond strength, and standardized relatable measurements [41]. It is suggested that the lower n-HA particle size (<100 nm) with spherical morphology, in comparison to micro HA, increases the exposed surface area for adhesion and mineral release [42]. In addition, a lower n-HA particle size also ensures adequate calcium and phosphate ion release (essential for remineralization) while retaining excellent mechanical properties [43], although this particular aspect was not investigated in our study. In the present study, <100 nm n-HA particles enhanced the μTBS of HAA-5% group as compared with the CEA group (without n-HA particles). Another probable reason for the improved μTBS for HAA-5% is its ability to biomineralize with the collagen network of tooth tissue [44], resulting in enhanced remineralization and bond integrity. However, it should be noted that the μTBS of HAA-10% was lowest among all groups, suggesting a compromise in dentin interaction and penetration, hybrid layer formation, mechanical properties, and dentin wetting. A study by Wagner et al. reported a decrease in adhesive bond strength with an increase in filler wt % of >10%, which was due to an increase in the viscosity of the material [45]. In the present study, agglomerate formation in HAA-10% specimens was detected in SEM micrographs; such clusters can lead to defects in the bonding complex concentrating applied stresses, decreasing bond strength [34]. The μTBS outcomes for HAA-5% are in agreement with the findings of Leitune et al., as they demonstrated an improvement in bond strength with the inclusion of n-HA in the experimental adhesive resin in comparison to control group [21]. In order to mimic the oral cavity’s dynamic conditions, bonded dentin–adhesive beams were thermocycled to depict bond durability with temperature changes encountered in an in vivo environment. Earlier, Helvatjoglu-Antoniades et al. demonstrated that thermocycling could reduce the bond strength of dentin adhesives [46]. Their findings are in conformity with the present study, as all adhesive specimens (CEA, HAA-5%, and HAA-10%) showed a decrease in μTBS due to TC [46].

In the present study, the uniform mixing and dispersion of n-HA particles within the experimental adhesive, as shown by the SEM images, is critical to positively alter the properties of dentin adhesive. If the particles are not properly diffused in the adhesive, it could lead toward observation of gaps or cracks, hindering the optimal performance of the adhesive [47]. It should also be noted that numerous resin tags with varying depths were observed on SEM micrographs with a similar distribution for CEA and HAA specimens, indicating an optimal removal of smear layer and adequate adhesive penetration at the dentin interface. This is usually the case when the performed etching is sufficient to remove the smear layer [47]. However, it should be noted that the resin tag depth in the tubules does not necessarily impact the bond strength and intactness of the adhesive, as reported by Anchieta et al. [48].

Micro-Raman spectroscopy is considered to be a vibrational spectroscopic diagnostic technique used to study the polarization of molecules [49]. We employed Micro-Raman spectroscopy to study n-HA as a bonding component in the hybrid layer, as this technique involves scattering instead of absorption; therefore, specimen of any thickness can be visualized without any destruction [50]. In our study, n-HA demonstrated intense phosphate and carbonate peaks, whereas for the HAA-5% group, although these peaks were visible, their intensity was decreased. The plausible reason of this particular finding could be that there is a transitional change in the intensity of the Micro-Raman bands when the minerals are replaced with resin, as reported previously [46]. Our study results are in line with a previous study as the peak intensity for phosphate and carbonate groups decreased for HAA-5% group [51].

The representative FTIR spectra in our study demonstrated a characteristic split band and sharp phosphate band for n-HA particles. Then, the same particles were added to the experimental adhesive to achieve HAA-5% and HAA-10% adhesive. There was a linear inverse relationship between the addition of n-HA particles and DC observed in the study. The DC of HAA-5% and HAA-10% group decreased compared with CEA group, and this finding is contradictory to Zhang and Wang, who reported that the DC increases when HA particles are added to the experimental adhesive [52]. The possible reason for this finding could be attributed to the fact that they used low HA concentration (1–4 wt %) in their study [52], in contrast to the 5-10 wt % used in the present study. The n-HA particles are opaque in nature to the visible light and can affect polymerization adversely [53]. Therefore, a high n-HA concentration in experimental adhesive could provide a less favorable medium for the light penetration and polymerization of the monomers to polymers, hence, leading toward less DC. Therefore, it is suggested that n-HA should be added to the adhesive with a diluent that could regulate its increased viscosity, leading toward more DC. Another reason could be that the addition of a higher amount of HA in the adhesive causes higher viscosity, leading toward a resultant low DC [21], although in the present study, the viscosity of the adhesives was not investigated.

In the present study 10 wt % HAA incorporation in experimental adhesive showed an agglomeration of particles with lower μTBS and DC, and these findings could critically compromise the dentin adhesion overtime. Treatments are available to improve the interface between the nanoparticles and the surrounding polymer to improve filer dispersion, including the addition of reactive groups, silane modifications, and polymer grafting Moreover, the addition of diverse ratios of n-HA in the adhesive could assist in establishing precise μTBS values in the dentin [27]. Therefore, further studies investigating the influence of incorporating surface-modified HA nano-spheres at higher concentrations and diverse ratios on mechanical properties, adhesive bonding, and the degree of conversion are recommended. In addition, the leaching of HA particles with possible cytotoxic effects such as free radical generation is a possible concern. As the biological interaction of HA nanoparticles with cell types and tissues were not assessed in the present study, future studies on the organic response of surface-modified HA particles would be revealing.

## 5. Conclusions

Within the limitations of the study, incorporated n-HA spheres uniformly dispersed in the experimental adhesive to exhibit dentin interaction and the formation of a hybrid layer with resin tags at varying dentin depths. HAA-5% demonstrated superior μTBS as compared with HAA-10% and control adhesive. The degree of conversion for HAA-5% was comparable to the control adhesive; however, HAA-10% was the lowest. Therefore, the inclusion of 5 wt % n-HA could improve the adhesive’s mechanical properties as it proved to be promising filler in our study and could have practical applications in clinical dentistry.

## Figures and Tables

**Table 1:** Mean and SD of microtensile bond strength and failure modes among the tested study groups.

	μTBS (MPa) (Mean ± SD)			Failure Mode Analysis (%)		
Group (*n* = 10)	NTC	TC	*p*-Value *	Adhesive	Cohesive	Mixed
CEA	27.36 ± 2.80 ^a A^	-	<0.01	80	0	20
	-	21.71 ± 3.85 ^a B^		100	0	0
HA (5%)	32.50 ± 3.47 ^bA^			60	20	20
	-	25.31 ± 3.84 ^a B^		80	0	20
HA (10%)	24.41 ± 3.72 ^a A^			70	0	30
	-	17.60 ± 4.36 ^b B^		100	0	0

TC: thermocycling, NTC: no thermocycling, GOA: graphene oxide adhesive, CA: control adhesive, * ANOVA. Dissimilar small alphabets in same column indicate statistical significance. Dissimilar capital alphabets in row (same group) indicate statistical significance.

**Table 2:** Degree of conversion (%) of hydroxyapatite-modified experimental adhesives and control specimens.

Group	Degree of Conversion (Mean ± SD)	Tukey (*p* < 0.05)
CEA	62.3 ± 5.2	A
HA (5 %)	58.1 ± 4.8	A
HA (10 %)	51.8 ± 5.6	C

HA: hydroxyapatite-modified experimental adhesive, CEA: control experimental adhesive. Dissimilar uppercase letters indicate statistical significance.
